# Rating the "Raters": Legal Exposure of Trustmark Authorities in the Context of Consumer Health Informatics

**DOI:** 10.2196/jmir.2.suppl2.e8

**Published:** 2000-09-13

**Authors:** Nicolas P Terry

**Affiliations:** ^1^ Professor of Law and Co-Director, Center for Health Law StudiesSaint Louis UniversityUSA

## Abstract

There are three areas of potential legal exposure for an organization such as a trustmark authority involved in ehealth quality rating. First, an ehealth provider may make a complaint about negative or impliedly negative ratings rendered by the ratings body (false negative). Typically, a negative ratings complaint would rely on defamation or product disparagement causes of action. In some cases such complaints could be defended on the basis of absence of malice (US). Second, the rating body might render a positive rating on ehealth data that a third party allegedly relied upon and suffered injury (false positive). While the primary cause of action would be against the ehealth data provider, questions may arise as to possible liability of the trustmark authority. For example, some US liability exposure is possible based on cases involving the potential liability of product warrantors, trade associations and certifiers or endorsers. Third, a ratings body may face public law liability for its own web misfeasance. Several risk management approaches are possible and would not necessarily be mutually exclusive. These approaches will require careful investigation to assess their risk reduction potential and, in some cases, legislation.

**Full paper published as:**

Terry NP. Rating the "Raters": Legal Exposure of Trustmark Authorities in the Context of Consumer Health Informatics. Journal of Medical Internet Research 2000;2(3):e18. (http://www.jmir.org/2000/3/e18/)

